# A Benchmark Study of Protein–Fragment Complex Structure Calculations with *N*MR^2^

**DOI:** 10.3390/ijms241814329

**Published:** 2023-09-20

**Authors:** Felix Torres, Gabriela Stadler, Witek Kwiatkowski, Julien Orts

**Affiliations:** 1 Institute of Molecular Physical Science, Swiss Federal Institute of Technology, ETH-Hönggerberg, 8093 Zurich, Switzerlandgabriela.stadler@phys.chem.ethz.ch (G.S.); witold.kwiatkowski@phys.chem.ethz.ch (W.K.); 2 Department of Pharmaceutical Sciences, Faculty of Life Sciences, University of Vienna, Josef-Holaubek-Platz 2, 1090 Vienna, Austria

**Keywords:** *N*MR^2^, NMR spectroscopy, drug design, complex structure, fragment, FBDD, structure–activity relationship

## Abstract

Protein–fragment complex structures are particularly sought after in medicinal chemistry to rationally design lead molecules. These structures are usually derived using X-ray crystallography, but the failure rate is non-neglectable. NMR is a possible alternative for the calculation of weakly interacting complexes. Nevertheless, the time-consuming protein signal assignment step remains a barrier to its routine application. NMR Molecular Replacement (*N*MR^2^) is a versatile and rapid method that enables the elucidation of a protein–ligand complex structure. It has been successfully applied to peptides, drug-like molecules, and more recently to fragments. Due to the small size of the fragments, ca < 300 Da, solving the structures of the protein–fragment complexes is particularly challenging. Here, we present the expected performances of *N*MR^2^ when applied to protein–fragment complexes. The *N*MR^2^ approach has been benchmarked with the SERAPhic fragment library to identify the technical challenges in protein–fragment NMR structure calculation. A straightforward strategy is proposed to increase the method’s success rate further. The presented work confirms that *N*MR^2^ is an alternative method to X-ray crystallography for solving protein–fragment complex structures.

## 1. Introduction

**Figure 1:**
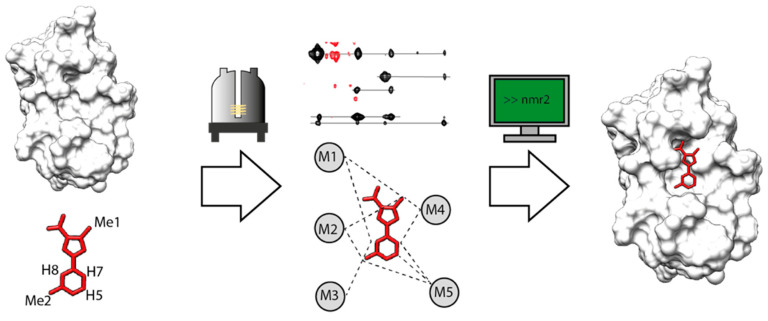
*N*MR^2^ workflow. An NMR sample with ^13^C,^15^N labeled protein and unlabeled ligand is prepared. F_1_-[^13^C,^15^N]-filtered-[^1^H,^1^H]-NOESY experiments are measured optimally with different mixing times. A network of semi-ambiguous distance restraints is built from the assigned ligand’s protons and the anonymous protein’s methyls NOESY cross-peak intensities. The semi-ambiguous distance restraints are used by *N*MR^2^ to calculate the correct protein–ligand complex structure.

Fragment-based drug discovery (FBDD) has proven valuable during the last 20 years for generating lead compounds for a broad range of protein targets [1,2,3]. The swift market entry of vemurafenib, the first approved FBDD-derived drug, established a gold standard in drug discovery research. Indeed, it took only six years to go from the fragment to the approved drug [4]. The standard approach starts with screening a library of thousands of fragments, ca < 300 Da, onto a validated target. Small compounds enable us to probe a more significant portion of the chemical space at an early stage compared to using more complex molecules [5,6]. Considering that the whole chemical space for small molecules (<1000 Da) is 10^60^, a library of 10^7^ molecules would represent only an infinitesimal portion of it [7,8]. However, the chemical space of small fragments (<160 Da) is estimated to be ca. 14 million compounds [9]; therefore, a library of 5k compounds achieves a higher chemical space coverage as compared to a library of larger molecules. The screening can be performed with different biophysical techniques such as X-ray crystallography [10], surface plasmon resonance [11], or, more recently, DNA-encoded libraries [12]. The hit molecules collected from this first cycle should be validated using an orthogonal method, such as NMR ^15^N-HSQC experiments [5]. The final step before engaging the hits into the medicinal chemistry pipeline is often to obtain structural information. The structure of a protein–ligand complex helps to understand the key features of the interactions and facilitates the design of a lead compound by growing or linking the initial fragments. The gold-standard method for protein–fragment structure characterization is X-ray crystallography [6,13]. However, due to the weak affinity of most fragments, co-crystallization is particularly challenging. To address this issue, fragments are soaked into the crystallized protein [14]. Nevertheless, many systems are incompatible with this approach. This can be the case when the protein does not crystallize, or the crystal is impervious to soaking. Moreover, the crystallized protein often adopts an energetically minimized landscape, which can significantly differ from the solution state. Consequently, key interactions between the ligand and the protein can be hindered depending on the crystal form [15]. In addition, the crystallization buffer used for the soaking maintains the crystal integrity, and this could be incompatible with the required fragment concentration (>10 mM) [16]. On the other hand, NMR spectroscopy is particularly suitable for the observation and the structural characterization of weak interacting systems in solution. Nevertheless, the time-consuming protein signal assignment step is a severe hurdle for using NMR in protein–fragment complex structure determination, especially when many systems need to be elucidated. The NMR Molecular Replacement (*N*MR^2^) method has already demonstrated the possibility of reducing the time needed to calculate protein–ligand complex structures from months to days of analysis [17]. The method employs a simulated annealing algorithm that calculates the complex structures from semi-ambiguous intermolecular distance restraints obtained from NMR. The restraints are defined as semi-ambiguous because the ligand’s signals are assigned while the protein methyls’ signals are not (Figure 1). *N*MR^2^ is a versatile method that has been used to calculate the 3D structure of different types of complexes, such as strong or weak interacting [17], and, more recently protein–fragment complexes [18]. Thanks to its reduced timeline, *N*MR^2^ is a serious alternative to X-ray crystallography for the structural determination of protein–ligand complexes, especially in the case of weak binders such as fragments. Moreover, as opposed to other fast NMR-based drug design methods, such as chemical shift perturbations, the method can entirely rely on experimental data. To establish a general methodology for using *N*MR^2^ in the protein–fragment complex structure calculation, the *N*MR^2^ algorithm has been benchmarked with the SERAPhic library [19]. The results from this benchmark provide insights into the complex interplay between the distance restraints tolerance, the amount of information contained in a sparse NMR dataset, the ligand and the binding site topology, and the result of *N*MR^2^ calculations. Finally, we propose a strategy to standardize the analysis of structural data and reinforce success in FBDD.

## 2. Results

In the context of drug-like ligands (i.e., MW > 350 g/mol), on average, 19.6 intermolecular distance restraints [17] are used to calculate the complex structures, while only 12.3 in the case of fragments [18]. This problem has been previously identified and addressed using partial assignments (PA) extracted from complementary NMR experiments [18]. The comparison of experimental *N*MR^2^ studies with drug-like molecules against studies with fragments suggests that *N*MR^2^ performs poorly when only a few (<13) distance restraints are provided, whereas the introduction of protein partial assignment is sufficient to significantly improve the performances. Therefore, testing the algorithm with a larger protein–fragment complexes dataset is necessary to fully understand the drivers of *N*MR^2^ success in the fragment context. For this reason, a proper benchmark has been conducted with a library dedicated to protein–fragment structures [19]. The dataset comprises 39 monomeric protein–ligand complex structures providing a variety of ligand and binding pocket sizes and representing a wide range of different pocket/ligand size ratios (Table S1). This topological variety is essential to show that the *N*MR^2^ method is versatile and capable of coping with various binding sites.

### 2.1. NMR^2^ Performances with Exact Distances

First, the exact distance restraints were provided for structure calculation, i.e., no tolerance was applied to estimate the performance of the *N*MR^2^ algorithm with exact data. This first round of calculations provided 35 correct ligand poses with a TFC > 0.2 out of 37, corresponding to 95% success. The initial dataset was composed of 39 structures, but two structures, with PDB codes 2BRT and 1S5N, were discarded for the reasons detailed below in the section on binding site topology. We found that the average heavy atom rmsd of the *N*MR^2^ calculated structures compared to the X-ray reference structures was 0.88 Å, which is significantly below the consensual value of 2 Å used to admit a correct ligand pose in the molecular modeling community [20,21]. Moreover, the two complex structures, which featured a TFC < 0.2 and were considered failed (PDB codes: 2FGQ and 2P1O), still ranked the right ligand pose within the ten best.

The TFC of the two structures could be significantly increased by introducing partial assignments to the *N*MR^2^ calculations as described in the material and method section. With the partial assignment, the TFCs of 2FGQ and 2P1O increased from 0.13 to 1.31 and from 0.16 to 3.4, respectively. Therefore, using partial assignments increased the success rate of *N*MR^2^ to 100%.

### 2.2. Conditions on the Binding Site Topology

**Figure 2:**
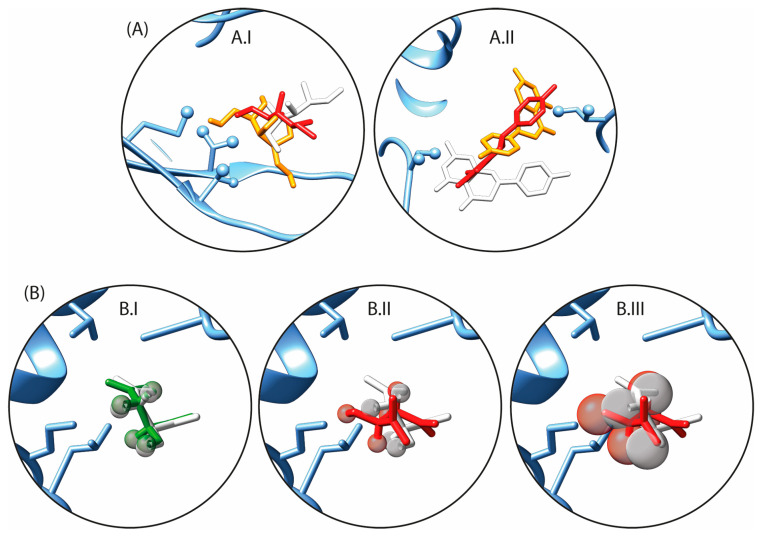
(**A**) Binding site of the non-converging *N*MR^2^ complex structures, (**A.I**) PDB code 1S5N (**A.II**) PDB code 2BRT. The X-ray crystal structure references are depicted in white, and the wrong ligand poses ranked as the first two best outputs from the NMR^2^ algorithm are colored in orange and red. (**B**) Schematic representation of the effect of the distance restraints tolerance on the calculated ligand poses. The reference ligand pose from the X-ray structure is depicted in white (PDB code 2FGQ). The distance restraint tolerances are illustrated with grey spheres with a radius corresponding to 10% or 20% of the restraints. (**B.I**) Overlap of the best predicted pose, depicted in green, with the X-ray structure, depicted in white, when the distance restraints tolerances are set to 10%. (**B.II**) Same as (**B.I**) but showing an incorrectly predicted pose, depicted in red. (**B.III**) Same as (**B.II**) but with distance restraints tolerances set to 20%.

Two complex structures, 2BRT and 1S5N, exhibit particular binding site topology. The detailed examination of the binding sites for these two structures revealed distinct topologies that appear incompatible with *N*MR^2^. The 1S5N complex binding site carries the three methyl-containing residues of its binding site on only one side of the binding pocket (Figure 2A.I). The 2BRT complex contains only two residues that carry methyls (Figure 2A.II). Consequently, these two binding sites will provide unresolved ligand poses, and therefore, 2BRT and 1S5N were excluded from the rest of the *N*MR^2^ calculations. As a guideline, the binding site should exhibit at least three methyl-containing residues relatively evenly distributed among the binding pocket. This was already suggested in the first publication regarding the *N*MR^2^ method, where the global positioning system (GPS) analogy was made [17]. The distribution of the methyl groups in the binding site should ideally be even. The determination of the position of the ligand on the protein surface by *N*MR^2^ is based on trilateration, namely only based on distance restraints from unknown protein methyl groups to ligand protons. If the methyl groups are degenerated or if there is an inherent symmetry of the distance restraints, the *N*MR^2^ method will provide ambiguous outputs. While potentially dangerous, it is, in principle, possible to artificially create the optimal binding site topology by introducing mutations, but this could heavily bias the drug design optimization steps [22]. Therefore, nearly degenerated methyl positions or insufficient methyl probes should be avoided when using *N*MR^2^.

### 2.3. Impact of the Distance Restraints Tolerance and Protein Partial Assignment on the NMR^2^ Convergence

Second, a tolerance of 10% or 20% was applied to the in silico distance restraints. Experimentally, distance restraints are obtained from the normalized NOE cross-peak intensities build-up curves, and tolerances may be applied to account for errors [23].

**Figure 3:**
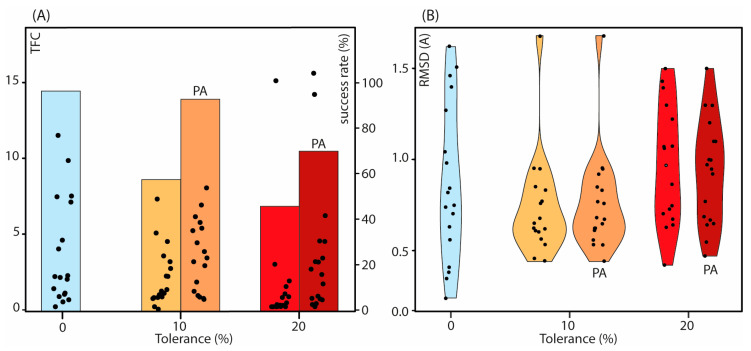
(**A**) TFC distribution for each *N*MR^2^ structure calculation, represented by dots and reported on the left y-axis, at different levels of tolerance applied to the distance restraints reported on the x-axis. The colored bar plot represents the success rate of the *N*MR^2^ calculations. Cyan, light orange, orange, red, and magenta correspond to tolerances of 0%, 10%, 10% with PA, 20%, and 20% with PA, respectively. PA means that the calculations that were run with partial assignments. (**B**) Violin plots of the rmsd (Å) values calculated between the ligand poses from the *N*MR^2^ structures versus the X-ray structures at different tolerance levels, where the individual values are displayed as dots. Cyan, light orange, orange, red, and magenta correspond to tolerances of 0%, 10%, 10% with PA, 20%, and 20% with PA, respectively. The distributions for the rmsd and the TFC use only the values of the structures that converge in all the different conditions.

The introduction of distance tolerance negatively affects the efficacy of the *N*MR^2^ calculations. This can be observed in Figure 3A where the *N*MR^2^ success rate drops from 95% when no tolerance is applied to 46% when ±20% tolerances are applied. The effect of increasing distance restraints tolerance on the TFC is well illustrated when comparing Figure 2B.II,B.III, where it is shown that large tolerances compared to the size of the ligand will prevent accurate positioning of the ligand in the binding site. In contrast, smaller tolerance will clash with the distance restraints. Consequently, different poses become possible with no significant target function penalty (Figure 2B.III). From the complete set of *N*MR^2^ structure calculations, the TFC drops upon the increase of the distance restraints tolerance, in some cases below the 0.2 threshold, leading to ambiguous structures (Table 1).

Subsequently, the effect of the distance restraints tolerance on the rmsd, calculated between the *N*MR^2^ prediction and the reference structure, has been evaluated. The distribution of the rmsd values with various distance restraint tolerances is plotted in Figure 3B and does not draw any clear tendency. The rmsd distribution of the *N*MR^2^ outcomes using 10% tolerance on the distance restraints is less scattered than the rmsd distribution when using a 0% or 20% tolerance. The corresponding average rmsd is 0.74 ± 0.29 Å versus 0.87 ± 0.43 Å and 0.97 ± 0.32 Å when using a 10%, 0%, and 20% tolerance on distance restraints, respectively. The lower value of the rmsd was assessed with a Student’s statistical *t*-test and interpreted at risk α = 0.05. We found no significant or little differences between the rmsd distributions (see Supplementary Information Table S2). Regardless of the amount of tolerance applied to the distance restraints, the average values of the rmsd (Table 1) are significantly below 2 Å, the consensus in the structure prediction and molecular modeling community to accept a pose as correct.

Introducing a partial assignment into the calculations with 10% or 20% tolerance improved the TFC values and consequently the success rate (Figure 3A). This is readily explained by looking at how the partial assignment operates. By fixing some of the assignment possibilities, the number of degrees of freedom decreases, and the TF penalty increases in the case of a wrong pose. Table 1 reports an increase in the TFC upon the introduction of partial assignments into the calculation. Therefore, partial assignments are highly recommended for the *N*MR^2^ structure calculations of protein–fragment complexes.

On the other hand, the introduction of partial assignments did not bring about significant changes in the individual rmsd values (Table 1, Figure 3B). This suggests that the partial assignments mainly influence the convergency of the *N*MR^2^ calculations rather than the quality of the converged structures.

### 2.4. Distance Restraints Network Topology Is Critical for a Successful NMR^2^ Structure Calculation

The ligand size, the binding site shape, and the number of intermolecular distances may also influence the outcome of the *N*MR^2^ method. Intuitively, a tight protein cavity with respect to the ligand, or pocket/ligand size ratio close to 1 (Table S1), will reduce the conformational space search significantly and improve the convergency of the *N*MR^2^ method. Generally, the smaller the ligand, the fewer the number of protons, decreasing the number of distance restraints. However, the influence of the ligand size is best rationalized with the topology parameters (r_l_ and r_w_), defined in the Material and Methods section, which address the characteristic geometry of the ligand in the binding pocket while considering its length, width, and the intermolecular distance restraints average. The topology parameters provide a rudimentary but efficient insight into the binding site topology.

When plotting the successful and unsuccessful *N*MR^2^ calculations according to the number of distance restraints versus the sum of the topology parameters, r_l_ + r_w_, we observe two clusters along the topological parameter axis (Figure S1). The *N*MR^2^ structure calculations mostly fail when the parameters r_l_ + r_w_ drop below 2, but are mostly successful when r_l_ + r_w_ is above 2. Interestingly, the amount of distance restraints, reported on the y-axis of Figure S1, does not seem to be a critical parameter, given it is above 12.

**Figure 4:**
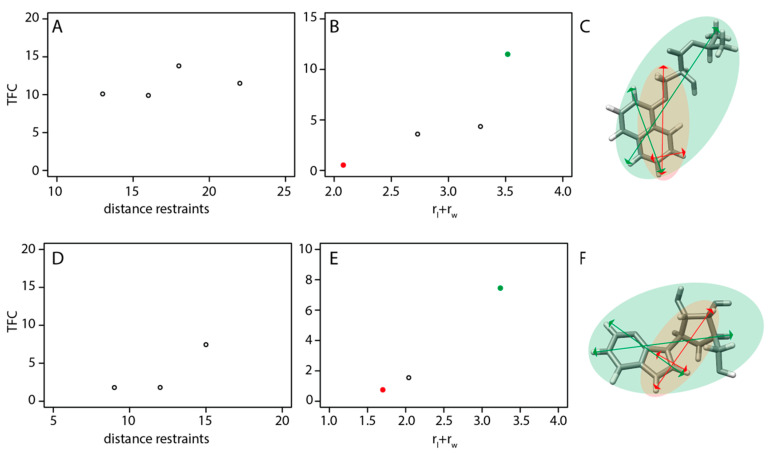
(**A**,**D**) Scatter plot of the TFC on the y-axis versus the distant restraints number represented on the x-axis. (**B**,**E**) Scatter plot of the TFC, on the y-axis, against the sum of the topology parameters reported on the x-axis. (**C**,**F**) Schematic illustration of the length and width vectors of the active protons of the fragments depicted in (**C**,**F**). The red and green arrows describe the topologies corresponding to the red and green dots in (**B**,**E**). The active protons are the ones involved in at least one distance protein–ligand restraints. (**A**,**B**) refer to the molecule 1H46 depicted in (**C**), and (**D**,**E**) to the molecule 2FF2 shown in (**F**).

The influence of the number of distances on the success of the *N*MR^2^ calculations is further investigated. Figure 4 shows the effect of the number of distance restraints and ligand topology on the TFC for two arbitrarily chosen structures (PDB codes 1H46 and 2FF2). A low TFC means that the *N*MR^2^ structure calculations did not converge to a single solution, but potentially converged to multiple ones, and is considered a failure, even if the actual structure is among the top-ranked poses.

As observed in Figure 4A, when the distance restraints number decreases from 22 to 13, the TFC remains constant. In the case of 2FF2, the TFC drops when the number of distance restraints drops below 15 but stays at a minimum value of 1.7, which is above the minimum TFC of 0.2 used as a threshold (Figure 4D). Interestingly, when the protein–ligand distance restraints are missing, relevant small molecule topology parameters for the *N*MR^2^ structure calculation can be affected. The withdrawal of distance restraints affects the apparent topology as it is illustrated in Figure 4C, where the green area represents the topology parameters of 1H46 with 22 distance restraints (Figure 4F, 15 distance restraints for 2FF2), and the red area the topology parameters of 1H46 with only the 12 shortest distance restraints (Figure 4F, nine distance restraints for 2FF2). In this case, the sum of the ligand topology parameters decreases, as shown in Figure 4B,D, with the green and red dots corresponding to the green and red area in Figure 4C,F, respectively, and the TFC drops significantly. The TFC of 1H46 decreases to 0.7 (Figure 4B) and the TFC of 2FF2 to 0.4 (Figure 4E). In summary, this suggests that the ligand topology parameters are more critical than the distance restraints number; in other words, the distribution of the protein–ligand distance restraints on the ligand structure is crucial for the success of *N*MR^2^.

**Figure 5:**
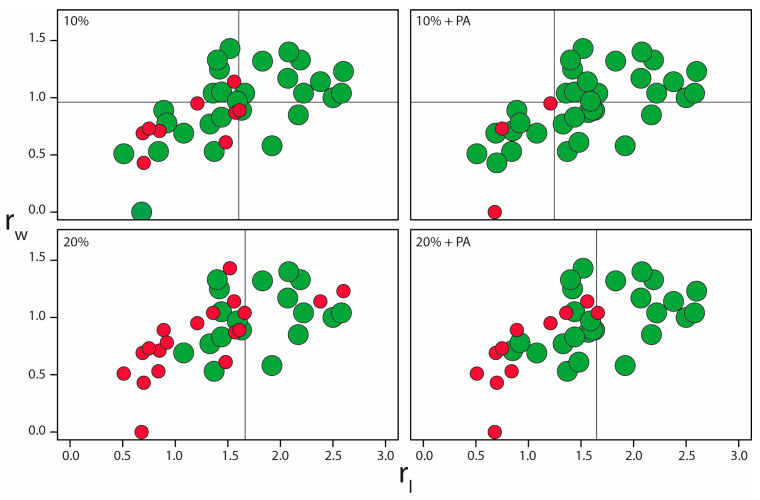
xy-scatter plots of the successful (green) and the unsuccessful (red) NMR^2^ calculations along the topology parameters r_l_ and r_w_. The different color codes correspond to the distance restraint tolerances applied (10% and 20%) and the use or not of partial assignments (PA).

The ligand topology parameters were further used to assess the relationship between the ligand–binding site topology and the success of the *N*MR^2^ calculation at different levels of distance restraint tolerances. Figure 5 draws the scatter plot of the r_l_ and r_w_ parameters at varying levels of tolerances with or without partial assignments, and each point corresponds to one protein–fragment complex. For the structures calculated with a 10% tolerance, a clear trend is observed between r_l_ or r_w_, and the success of the *N*MR^2^ calculations. This correlation is more substantial when partial assignments are introduced. When using 20% tolerance, the trend is observed only in the longest dimension, r_l_. The introduction of partial assignments in the *N*MR^2^ calculations improves the overall success with no fail structure calculation when r_l_ is above 1.7, suggesting that fragments need to be bigger to converge with *N*MR^2^.

This trend is also visible in Table 1, where the average values of topology parameters r_l_ and r_w_ are higher for the successful *N*MR^2^ calculations than those that fail. Adding partial assignments decreases the average topology parameters because more protein–ligand complexes containing smaller fragments converged in the *N*MR^2^ calculations. Overall, this suggests that the tolerance on the distance restraints is less critical for determining complex structures with high ligand topology parameters r_l,_ and r_w_. Furthermore, partial assignments improve the structure calculation of complexes with smaller ligands (i.e., with lower r_l_ and r_w_) even at 20% tolerance.

### 2.5. Computational Time

The calculations were performed using only 10 CPUs. However, the calculation time varied from a few minutes to 96 h, with a median calculation time of 11 min. No simple relationship could be established between the calculation time, the number of methyls, or the number of restraints. However, using partial assignments significantly decreased the calculation time, with a maximum calculation time of 38 h and a median calculation time of 10 min.

## 3. Discussion

The presented *N*MR^2^ benchmark demonstrates the possibility of calculating NMR-based protein–fragment complex structures. Despite the quality of the data provided by NMR, it is often considered a secondary technique in drug discovery due to the lengthy analysis required before structure calculation. However, *N*MR^2^ offers a promising alternative thanks to its reduced analysis time, especially in the context of FBDD campaign, where several protein–fragment structures must be derived. Furthermore, the expertise required to obtain such a structure is drastically reduced because the analysis is automated and straightforward. *N*MR^2^ can provide a structure in a few days, including the NMR data measurement and the structure calculation.

The data presented above enable the user to understand the critical parameters yielding to successful calculations and help establish a strategy that should be systematically applied for the *N*MR^2^ calculation of protein–fragment structures in the context of an FBDD campaign. The first important parameter is the binding site topology: proteins with fewer than three residues carrying a methyl group in the binding site or proteins with a non-isotropic repartition of these residues in the binding site are particularly challenging. A possible way to address these systems is to use single-point mutations with improved methyl density in the binding site.

Second, the distance restraint tolerance usually applied to experimental NOE significantly degrades the convergence of the calculations. Still, they do not lower the quality of the calculated structure, provided that a minimum of 15 distance restraints are present. In our hand, the following [^13^C,^15^N]-filtered-[^1^H,^1^H]-NOESY provides NOE cross-peaks of high quality [24], and normalizing NOE cross-peaks lead to higher-quality distance restraints [23] if the water suppression scheme does not skew the diagonal intensities. In that case, we recommend using deuterated solvent and a simple water presaturation during the mixing time. The attention of the reader is brought to the fact that a NOESY pulse sequence that keeps the possibility of quantitative analysis of diagonal- and cross-peaks and proper data analysis [23] should yield a tolerance of a maximum of 10% which was shown to significantly improve the algorithm convergence (Figure 3 and Figure 5). Moreover, the use of partial assignments significantly improved the calculation convergence by increasing the target function contrast (TFC) (Figure 5). These increased TFC values are explained by restricting the degrees of freedom within a given fragment-binding pocket topology. Introducing partial assignments only requires a maximum of a day of NMR measurement time when derived spectroscopically, or can be done via specific protein methyl group labeling schemes [25,26]. Using specific labeling schemes with protein perdeuteration provides the advantage of producing nearly perfect experimental intensities, free of spin diffusion artifacts, and a larger set of distance restraints since the NOEs span longer distances in perdeuterated protein. In addition, only the chosen amino acid methyl groups will exhibit NOEs to the ligand, already providing a partial assignment of amino acid types and reducing NMR peak overlap [27,28].

Fragments contain fewer protons than drug-sized ligands and thus provide fewer intermolecular distance restraints, with, on average, 19.6 intermolecular distance restraints for the drug-sized ligands and 12.3 distance restraints for fragments. Such a reduction in the number of restraints increases the accessible conformational space of the ligand in the binding site. Generally, it leads to a drop in the TF penalty for wrong structures because fewer restraints can be violated. We found that a TFC of 0.2 provided the best discrimination between wrong *N*MR^2^ poses and the correct ones when using perfect in silico data. This threshold is expected to increase when using experimental data, but it is unlikely to be smaller than 0.2.

To help rationalize and predict the success of *N*MR^2^ structure calculations with fragments, we introduced two topological parameters defined by the ligand characteristic sizes normalized by the average distance restraints. Long-distance restraints are less valuable for *N*MR^2^ because they tend to be fulfilled by many methyl groups from the protein receptor. The topological parameters increase when the average distance restraints decrease, capturing this effect. Furthermore, large compounds reduce the conformational space in the binding site via steric hindrances, positively affecting the conformational search. Large molecules are also less likely to exhibit molecular symmetries, chemically or magnetically equivalent protons that lead to symmetric ambiguous structural information detrimental to the *N*MR^2^ method. In that sense, a methyl-quinoline fragment is better suited for *N*MR^2^ than a heteroaromatic with a much smaller and roughly symmetric shape. This point is also taken into account by the topological parameters. We found that the higher the topological parameters, the better, allowing users to prioritize fragments versus others.

In summary, we recommend recording F_1_-[^13^C,^15^N]-filtered-[^1^H,^1^H]-NOESY (Figure S1) on samples containing doubly labeled (^13^C,^15^N)-protein and unlabeled fragments in 100% deuterated buffer, avoiding water suppression schemes that could strongly affect the spectra baseline or modify the diagonal signal intensities in the vicinity of the water signal [17,24]. We also recommend recording a ^13^C-ctHSQC and potentially an HCCH-TOCSY to collect partial assignment information, such as the amino acid type of the receptor methyl group resonances [18]. Alternatively, it is possible to use specific methyl labeling schemes with otherwise deuterated protein for this purpose [27,28]. Protein deuteration will also improve the quality of the NOE data. The NOE data should be processed and analyzed according to the previously reported protocols and fed to the *N*MR^2^ software (*N*MR^2^ software v1 from Orts et al. [29], Zurich, Switzerland). Priority should be given to the fragments having the highest topological parameters r_l_ and r_w_. If two fragments have the same topology parameters, the priority should be given to the one with the least internal degrees of freedom to ease the conformational sampling during the calculations. The methyl assignments obtained from the first *N*MR^2^ structure shall be propagated, in full or in part, to the following protein–fragment *N*MR^2^ structure calculation [18].

## 4. Material and Methods

The *N*MR^2^ workflow is depicted in Figure 1 and follows three main steps. First, a sample containing (^13^C,^15^N)-double-labeled protein and ligand is prepared, and a series of F_1_-[^13^C,^15^N]-filtered-[^1^H,^1^H]-NOESY are measured with different mixing times, typically from 20 to 120 ms (Figure S2) and preferably in D_2_O to avoid water suppression schemes that would potentially skew the diagonal peak intensities [24]. Second, the ligand signals are assigned in the NOESY spectra, and the protein methyl signals are arbitrarily labeled M_n_. Other protein functional groups can also be used, but the methyls are excellent probes because their resonance peaks are strong and still visible even for large receptors [27,28]. The ligand to protein methyl groups NOE cross-peak intensities are converted to the intermolecular semi-ambiguous distance restraints [17,23]. Finally, the semi-ambiguous intermolecular distance restraints network is passed to the *N*MR^2^ algorithm to calculate the protein–ligand complex structures [17,18,29]. *N*MR^2^ calculates the structures for different methyl assignment combinations using the CYANA structure calculation software 3.98 (www.cyana.org) [30].

The CYANA target function is used to rank the calculated structures. To confirm the validity of the best-ranked complex structure, the target function difference or target function contrast (TFC) with the first significantly different ligand pose (rmsd > 2Å) is calculated. The best-ranked structure is validated if the TFC is above 0.2 [18]. The choice of the minimum TFC value of 0.2 is based on the true positive and the false positive rates, as reported in Figure S3. With a minimum TFC value lower than 0.2, false positives start to appear, and with a minimum TFC value higher than 0.2, some true positives are not detected anymore. Tolerances may be applied on distance restraints, consequently reducing the TF. Indeed, when a tolerance is introduced, the distance from the calculated structure does not need to match the restraint exactly, but needs to be in a range defined by the lower limit and the upper limit distance restraint. For example, when using 10% tolerance, the upper and the lower limit distance restraints are, respectively, 10% higher and lower than the calculated restraints.

Partial assignment of the protein methyl groups can provide, at low cost, extra information, helping *N*MR^2^ to improve the accuracy of the predicted structure [18]. For example, an HCCH-TOCSY can inform whether two methyls belong to the same residue and identify the alanine and threonine residues due to their unambiguous patterns [31]. A ^13^C-ctHSQC can identify the methionine methyls if the constant time is fixed at 13.3 ms [32]. Further description of the partial assignment strategy is available in the Supplementary Information (Figure S4). In the present study, partial assignments were introduced as realistically as possible. We provided only additional data to the calculation as it would have been the case after performing HCCH-TOCSY and ^13^C-ctHSQC, followed by a straightforward analysis. For instance, the methyls belonging to a methionine are assigned to methionine without residue number; methyls belonging to a valine, leucine, or isoleucine are considered to belong to this group of residues without further information (see Supplementary Information).

The *N*MR^2^ calculations were performed on a simple cluster of 20 desktop computers with four CPUs each (HP Z240 with Intel(R) Xeon(R) CPU E3-1245 v5 @ 3.50 GHz and 32 GB) running under OS Ubuntu 20.0 and operated with a slurm workload manager. Each *N*MR^2^ calculation was run with 10 CPUs. The *N*MR^2^ simulated algorithm calls the CYANA software 3.98 to perform the structure calculation. The *N*MR^2^ calculations were performed in the binding site, with side chain flexibility set to 20 degrees for each dihedral angle. The intermolecular distance restraints were measured between the ligands’ protons and the proteins’ methyl carbons.

We selected the SERAPhic library for the benchmark, which contains high-quality protein–fragment X-ray structures [19]. The proteins, containing at least 200 amino acids, were chosen as representative of clusters at the homologous superfamily level of CATH to ensure a structurally and evolutionary diverse set of target receptors with minimal sequence identity (<21%). The library ensures the structural heterogeneity of the fragment by maintaining the Tanimoto similarity coefficient below 0.85. The similarity coefficient is defined as similarity = 1/(1 + distance), where the distance metrics are, in this case, calculated according to the Tanimoto methodology [33]. The Tanimoto methodology calculates the distance of two binary vectors characterizing the structures of the molecules. When this distance is close to 0, the similarity is close to 1. This metric is popular in medicinal chemistry and shows robust performances [34], ensuring the chemical diversity of the SERAPhic library. Furthermore, the properties of the fragments, such as the number of H-bond donors/acceptors, number of torsionals, number of rings, and log P, were shown to overlap well with the molecules in the DrugBank and KEGG COMPOUND repositories. Furthermore, the average pocket/ligand size ratio is 2.4, with a maximum and a minimum of 6.4 and 1, respectively (Table S1), covering a large panel of possible binding modes. Moreover, the median of all distances is 5.1 Å, consistent with the experimental ones obtained from prior studies, 4.4 Å [35]. The 0.7 Å markup for the distance restraints is because we consider here the carbon atoms of the protein to derive the in silico distances instead of protons. The dataset of 39 protein–fragment complex structures was prepared as follows: The intermolecular distances between the protons of the ligands and the methyls present in the binding site of the proteins were measured in silico. The assignments of the ligand’s protons were kept while the protein’s methyls were anonymized M_n_, where n is an arbitrary number assigned to each methyl. This later step constitutes the semi-ambiguous intermolecular distance restraints network of each complex.

The conformity of the complex structures calculated using *N*MR^2^ with the respective complex structures from the benchmark is verified following two criteria. First, the heavy atoms’ root mean square deviation (rmsd) must be below 2 Å. Second, the complex structures are overlaid and visually examined to ensure the orientation of the fragment in the binding site is the same.

In the following, the fragments are further described using topology parameters defined by the ratio of the characteristic internal dimensions of the ligand to the average intermolecular distance restraints length:

r_l_ = L_length_/<d>

r_w_ = L_width_/<d>

where L_length_ is the maximal distance between two protons within the ligand, or the length; L_width,_ the maximal ligand proton–proton distance orthogonal to the length vector. <d> is the average intermolecular distance restraints value for the complex. The length, L_length_, is defined as the longest distance vector that can be drawn between two protons belonging to the ligand, and the width, L_width_, is the longest vector orthogonal to the length vector that can be drawn between two protons belonging to the ligand. The mean distance is considered since longer distance restraints contain more tolerance than shorter ones. By doing so, the uncertainty is directly linked to the length of the intermolecular distance restraints. Figure S5 visually represents the topology parameters and their relationship with distance restraints tolerance.

## 5. Conclusions

In conclusion, *N*MR^2^ structure calculations are an attractive alternative to X-ray crystallography for protein–fragment structure calculation. While the pocket size is not critical for the success of *N*MR^2^ calculations, the ability of the fragment to build symmetry-breaking intermolecular distance restraints is critical. Another crucial point is the number of protein–ligand intermolecular distance restraints, which should be at least 12, while 19 or higher would be expected for a drug-like molecule. Moreover, special care should be brought to correctly measure and analyze NOE data to improve accuracy and precision. This is best done using the appropriate NOESY pulse sequence, deuterated buffer, and potentially partially deuterated protein with re-protonated methyl groups.

This benchmark provides a clear understanding of the route to be followed for successful protein–fragment complex structure calculation: (1) measurement and analysis of the fragments with the highest topological parameters; (2) introduction of partial assignments, if needed; (3) calculation of the first complex structure; (4) propagation of the assignments obtained from the first structure calculation to the further complex calculations.

## Figures and Tables

**Table 1:** Benchmark results overview.

Distance Restraints Tolerance	Success Rate	rmsd (Å)	TFC	Success: r_l_ r_w_	Fail: r_l_ r_w_
**0%**	95%	0.88 ± 0.42	2.56 ± 3.00	1.55 ± 0.58	0.98 ± 0.33
		0.87 ± 0.43 *	3.68 ± 3.50 *	0.91 ± 0.32	0.84 ± 0.16
**10%**	57%	1.05 ± 0.53	1.58 ± 1.99	1.63 ± 0.58	1.16 ± 0.41
		0.74 ± 0.29 *	2.16 ± 1.93 *	0.95 ± 0.33	0.78 ± 0.21
**20%**	46%	0.96 ± 0.27	0.98 ± 2.85	1.79 ± 0.44	1.25 ± 0.58
		0.97 ± 0.32 *	1.50 ± 3.48 *	1.00 ± 0.26	0.82 ± 0.33
**10% (PA)**	92%	0.85 ± 0.34	3.20 ± 3.80	1.57 ± 0.56	0.88 ± 0.29
		0.76 ± 0.26 *	4.51 ± 4.50 *	0.94 ± 0.28	0.56 ± 0.50
**20% (PA)**	70%	1.09 ± 0.51	2.29 ± 3.73	1.74 ± 0.49	0.99 ± 0.39
		0.93 ± 0.29 *	3.63 ± 4.45 *	0.99 ± 0.26	0.72 ± 0.34

* Calculated among the population of structures that converges for all conditions stated in the first column (PA means partial assignments).

## Data Availability

The data relative to the present study are available to the corresponding author on demand.

## Supplementary Materials

The following supporting information can be downloaded at: https://www.mdpi.com/article/10.3390/ijms241814329/s1.

## Abbreviations

CATH (Class Architecture Topology and Homologous superfamily); CPU (Central Processing Unit); CYANA (Combined assignment and dYnamics Algorithm for NMR Applications); HSQC (Hetero Single Quantum Correlation); ^13^C-ctHSQC (constant time HSQC); FBDD (Fragment-Based Drug Design/Discovery); KEGG (Kyoto Encyclopedia of Genes and Genomes); NMR (Nuclear Magnetic Resonance); *N*MR^2^ (*N*uclear Magnetic Resonance Molecular Replacement); NOE (Nuclear Overhauser Effect), NOESY (Nuclear Overhauser Effect SpectroscopY); PA (Partial Assignment); PDB (Protein Data Bank); RMSD (Root Mean Square Deviation); SERAPhic (Selected Fragment Protein Complexes); TF (Target Function); TFC (Target Function Contrast); TOCSY (Total Correlation SpectroscopY).
