# Supplementary material for: A Benchmark Study of Protein–Fragment Complex Structure Calculations with NMR2
Source: Int J Mol Sci. 2023 Sep 20;24(18):14329. doi: 10.3390/ijms241814329 (PMC10531959; doi:10.3390/ijms241814329)
Supplement: Supplementary file 1 [file ijms-24-14329-s001.zip › ijms-2570209-supplementary.pdf]

Supplementary information

A benchmark study of protein-fragment complex structure calculations with *N*MR²

Félix Torres [1], Gabriela Stadler [1], Witek Kwiatkowski [1], and Julien Orts [2]*

[1] Institute of Molecular Physical Science, Swiss Federal Institute of Technology, ETH-Hönggerberg, 8093, Switzerland;
[2] Department of Pharmaceutical Sciences, Faculty of Life Sciences, University of Vienna, Josef-Holaubek-Platz 2, 1090 Ausrtia
* Correspondence: julien.orts@univie.ac.at;

Table S1: Protein–ligand complex structure metrics relevant to the benchmark. The pocket size, ligand size and pocket/ligand size ratio.

pdb code	Protons anchors	Distance median	Pocket size A^3	Ligand size A^3	Pocket/ligand
2f6x	7	6.1	385	110	3.5
2bkx	10	5.3	200	200	1.0
2cix	6	4.8	135	75	1.8
1ynh	6	5.3	295	130	2.3
1wog	8	6.0	140	140	1.0
1k0e	7	4.4	340	150	2.3
1m2x	11	8.0	375	155	2.4
2ff2	5	4.7	250	195	1.3
2i5x	7	4.9	160	140	1.1
1h46	5	6.1	770	210	3.7
2iba	3	6.0	580	90	6.4
1tku	5	3.8	145	150	1.0
1m3u	3	4.1	215	105	2.0
1mlw	6	4.2	250	165	1.5
3c0z	9	7.2	300	90	3.3
1r5y	8	4.6	265	120	2.2
1y2k	7	4.1	240	220	1.1
2aie	8	4.2	285	165	1.7
2q6m	6	5.2	310	225	1.4
2zvj	6	4.9	140	195	0.7
2uy5	9	6.0	235	170	1.4
2j5s	11	4.1	210	115	1.8
2v77	10	4.1	295	155	1.9
1f5f	20	4.2	350	235	1.5
2gg7	7	7.5	350	165	2.1
2rdr	3	5.6	505	90	5.6
1ofz_b	8	4.5	490	105	4.7
1ofz_a	6	5.1	490	105	4.7
1pwm	9	5.2	350	190	1.8
2gvv	11	3.9	440	165	2.7
2hdq_a	3	6.5	460	90	5.1
2p1o	9	6.3	480	115	4.2
1uwc	7	4.4	240	150	1.6
1yv5	6	5.8	240	185	1.3
1e2i_b	9	4.1	290	145	2.0
1x07	3	5.1	280	150	1.9
2fgq	4	4.0	110	85	1.3
1s5n	5	4.8	450	100	4.5
2brt	8	4.8	190	195	1.0

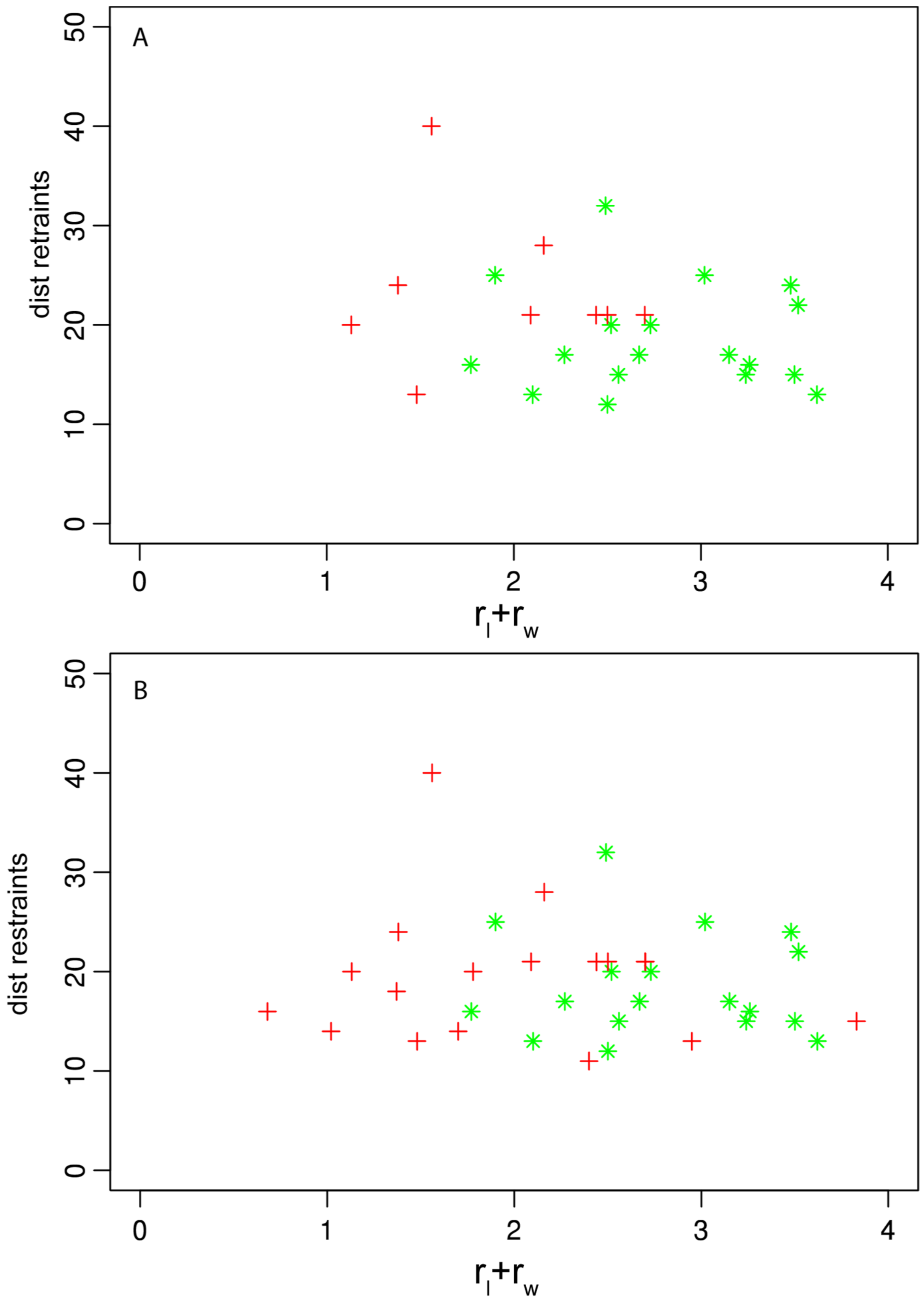

Figure S1: Distribution of the failed (red cross) and successful (green star) calculations mapped on the 2D plot representing the topology parameters sum r_l+r_w (x-axis) and the number of distance restraints (y-axis) with 10% (A) and 20% (B) tolerance.

F_1-[^{13}C,^{15}N]-filtered-[^{1}H,^{1}H]-NOESY

The acquisition of proton-proton cross-relaxation peaks to be processed into distance restraints is typically done by the F_1-[^{13}C,^{15}N]-filtered-[^{1}H,^{1}H]-NOESY experiment, depicted in figure S1. In a first instance the magnetization from the proton attached to ^{13}C or ^{15}N is filtered thanks to two consecutive x-filters (point *a* to *c*). The delays Δ1 and Δ2 are optimized for the suppression of the ^{1}H-^{13}C scalar coupling of 110 Hz and 140 Hz, respectively. These delays are optimized in concert with the carbon shaped pulses sp1 and sp2 which are designed to excite the broad range of carbon frequencies, according to the spectrometer magnetic field following the methodology described earlier by Zwalen et al. The filtered NOESY is usually recorded for a sample prepared in 100% D_2O so that the contribution of the amide protons is minim, therefore the elimination of the ^{15}N-^{1}H (90 Hz) coupled protons is sufficient in practice, despite the non-optimal delays. The t1 delay (*d* to *e*) is the indirect dimension evolution, it is kept between 360 (18.3ms) to 512 (26ms) points. The mixing time tm is kept between 15ms to 120ms to limit the contribution from spin diffusion, and several NOESY with different mixing times are recorded. During the mixing time (*e* to *f*) the magnetization is transfer from a proton to a proton through dipolar coupling. This coupling is time (tm) and distance dependent, this is why the resulting cross-peak intensity build-ups can be processed into distance restraints. In the end the pathway is summarized such as: ^{12}C-^{1}H → ^{12}C-^{1}H, ^{13}C-^{1}H. The arrow symbolized the mixing time, tm. The details for optimized acquisition parameters at different fields are available in Zwahlen et al, 1997.[1]

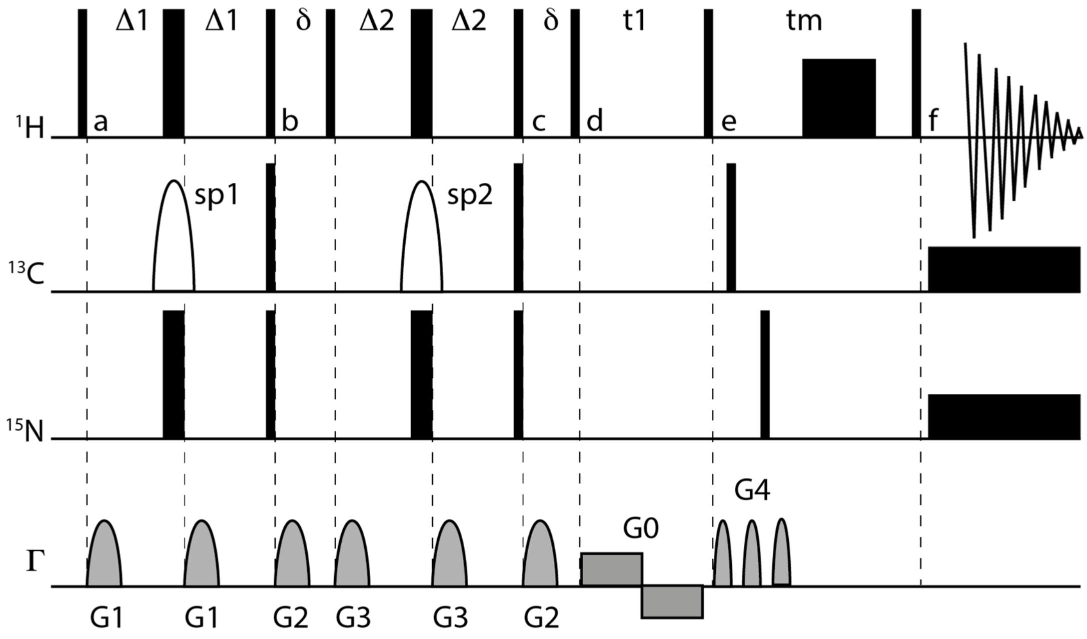

Figure S2: F_1-[^{13}C,^{15}N]-filtered-[^{1}H,^{1}H]-NOESY pulse sequence. The sharp dark rectangles are for 90° hard pulses and the broad dark rectangles are for 180° hard pulses. sp1 and sp2 design shaped pulse optimized to excite the broad range of carbon frequencies, they are both wurst pulses optimized for the desired magnetic field according to Zwalen et al. At a field of 700 MHz, sp1 is xxms; sp2 is xxms. Where the delays are at the fixed values optimized for proton-carbon coupling frequency selection. E.g. for 700MHz spectrometer: Δ1=0.685ms, Δ2=0.785ms, δ=0.200ms. Γ are the field gradients along the z-axis, with G0=2ms, G1=0.5ms, G2=1ms, G3=0.5ms, G4=1ms. The pulse during the mixing time tm is a trim pulse for water suppression at same power level as hard pulses and lasts 1 to 2ms. The indirect dimension acquisition is designated as t1.

Choice of the TFC threshold value based on the true positive and false positive rates

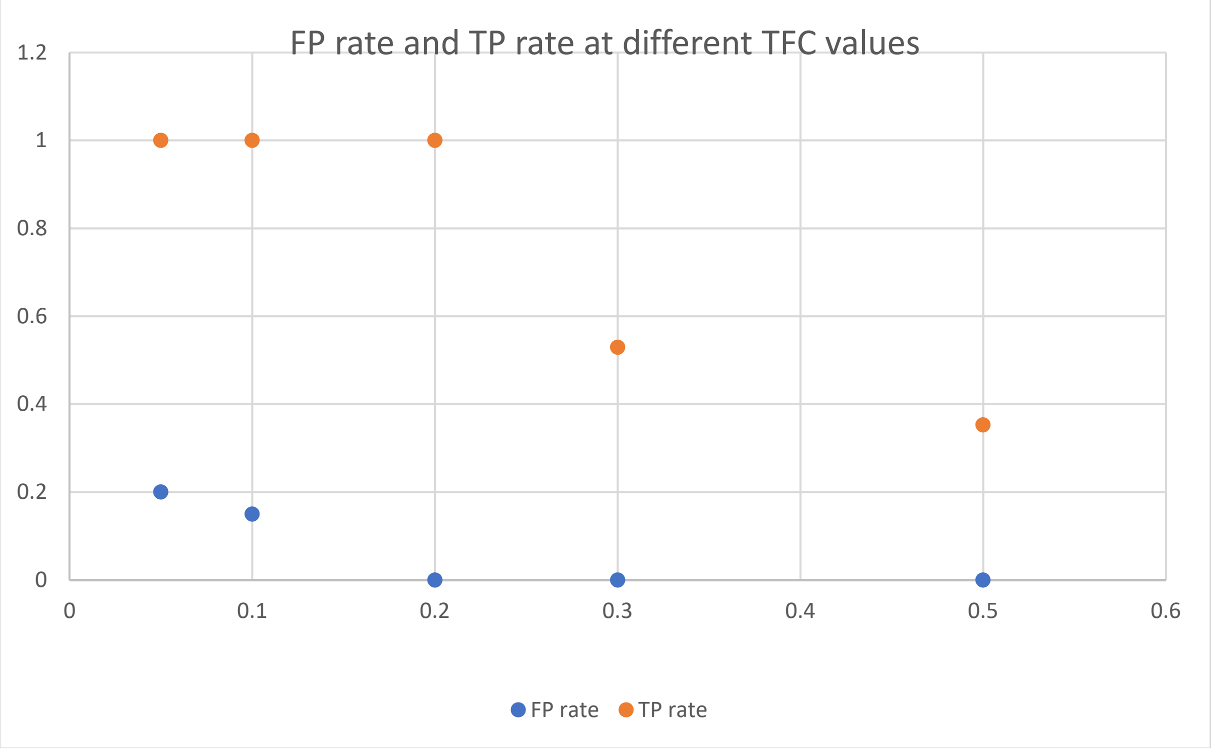

Figure S3: Evolution of the false positive (FP) and true positive (TP) rates at different TFC values. The threshold value of 0.2 is justified by a maximum number of TP and a minimum number of FP. The rates are calculated for the 37 structure calculations with 20% of tolerance applied to the distance restraints.

The FP rate is defined as: FP/N with N=FP+TN.
The TP rate is defined as: TP/P with P=FN+TP.
TN is for true negative and FN is for false negative.

Partial assignment strategy

The methyl identity can be readily obtained from a ^{13}C-constant time Heteronuclear Single Quantum SpectroscopY (^{13}C-ctHSQC) and HCCH-TOCSY NMR experiments.

We provide here the rational to refine/define the methyl identity based on these two experiments. In the case a methyl is identified as belonging to a certain type of amino-acid (i.e. threonine), if this amino-acid is present in only one copy in the binding site, it can be thus assigned directly. Otherwise, the identity of the methyl can be set specifying the type only, e.g. methyl in δ of a isoleucine.

Alanine: Easily recognizable because the methyl only correlated to a Hα in the HCCH-TOCSY.

Valine: Valine has a specific pattern in the HCCH-TOCSY. Two methyls, a single Hβ, and a Hα. However, depending on the signal to noise or the signal overlap, it can be difficult to distinguish from the leucine.

Leucine: Leucine has a specific pattern in the HCCH-TOCSY. Two methyls, two Hβ, a single Hγ and a Hα. However, depending on the signal to noise or the signal overlap, it can be difficult to distinguish from the valine.

Threonine: Looks like valine but with only one methyl. The, carbon frequency plane of the Hβ is downfield and the carbon frequency plane of the Hα is upfield, due to the hydroxy group on β-position. Moreover, the cross-peaks between the Hβ and the methyl in Cβ plane is of opposite sign, thus facilitating the identification.

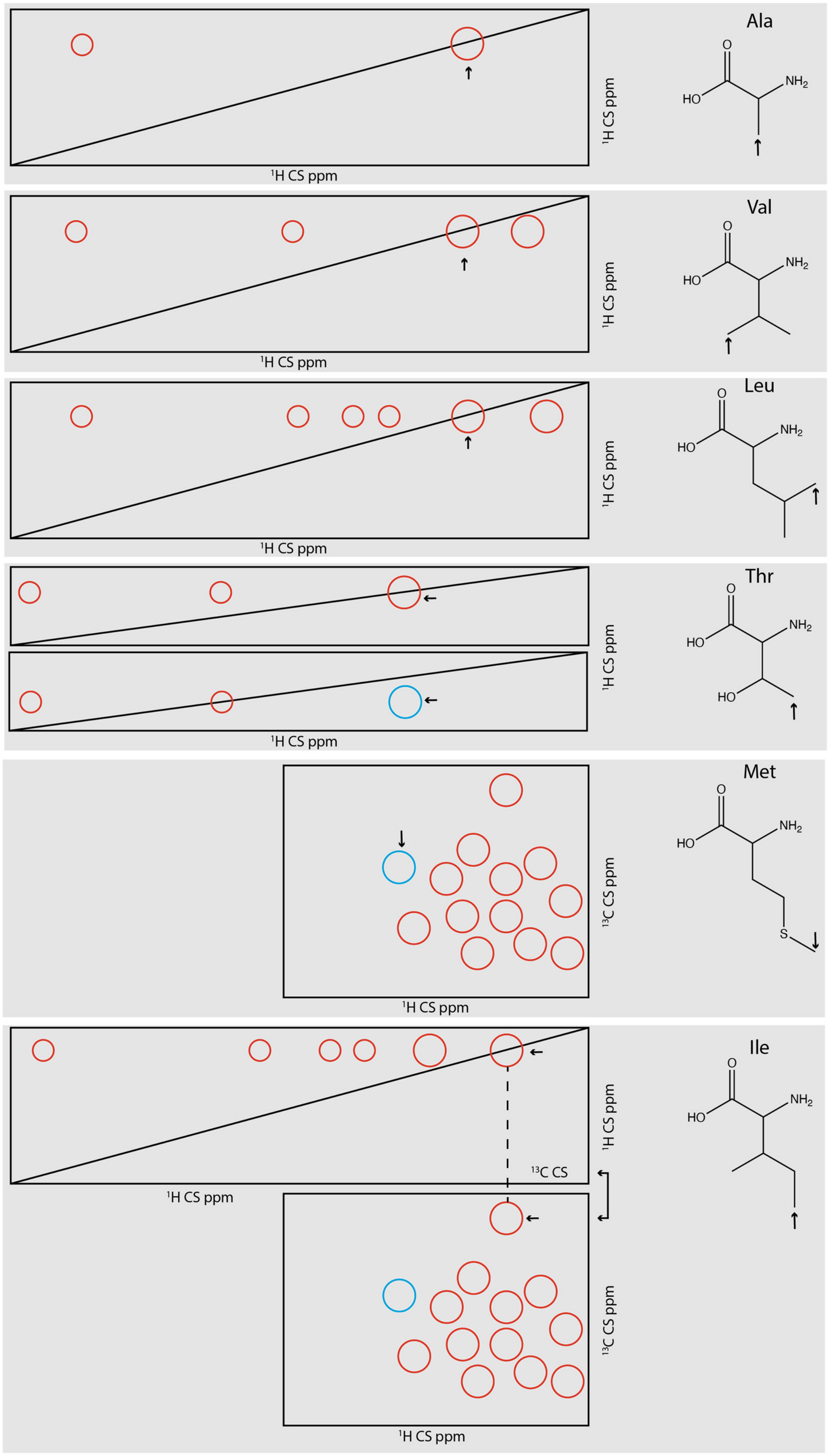

Figure S4: Pattern recognition strategy for the partial assignment of methyls prior to NMR[2] calculation.

Methionine: In the 13C-ctHSQC with a constant time delay of 13.3ms the methionine methyl has an opposite sign as compared to the other methyls. Furthermore, it is relatively downfield in comparison to usual methyl chemical shifts.
Isoleucine: The methyl in δ position is readily identified, thanks to its upfield carbon chemical shift as compared to the other methyls. The pattern is close to the one of leucine, but here it's the Hβ which is single, and the Hγ are two. The methyl in γis identified, thanks to its correlation to the methyl in δ.

Student t-test for the RMSD distributions

The Student t-test have been conducted to probe the difference between the RMSD distributions. The only distributions exhibiting statistically significant difference at risk $\alpha = 0.05$ were 10% tolerance and 20% tolerance (10-20%) applied to the distance restraints. This is explained by the relatively small standard deviation of the differences between the RMSD values.
The rmsd distributions for the calculations run using 0% and 10% tolerance showed no significant difference (p-value = 0.2032). The rmsd distributions of the calculations at 10% and 20% tolerance showed significant difference (p-value = 0.0063) suggesting an increase of the rmsd with higher amount of noise. However, no significant difference was shown while comparing the rmsd at 0% and 20% of distance restraints tolerance (p-value = 0.4381). The results of the Student t-test are reported in Table S2.

Table S2: Student t-test results for the RMSD distribution at different level of distance restraint tolerances.

Comparison	n	t	p-value	IC 95%	Diff mean	Diff SD
0-10%	17	1.3268	0.2032	[-0.0762,0.3313]	0.31	0.26
10-20%	17	-3.1404	0.0063	[-0.0734,0.3784]	0.29	0.23
0-20%	17	-0.7952	0.4381	[-0.3607, 0.1639]	0.39	0.33

Distance restraints number and topology parameters

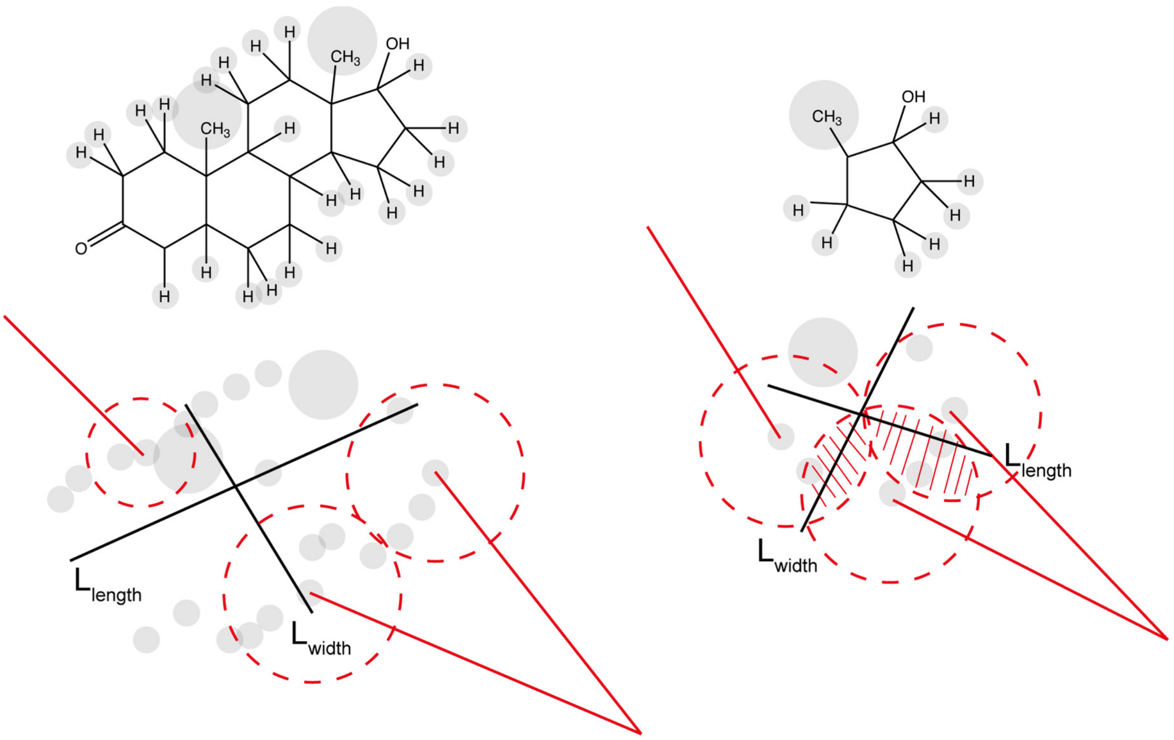

Figure S5: schematic representation of the topological parameters (L_{length} and L_{width}) for a big (left) and a small (right) molecule. The red lines represent the protein–ligand semi-ambiguous distance restraints and the dashed red circles are the tolerance on the position of the small molecule proton. The overlapping surfaces are hatched.

References

[1] C. Zwahlen, P. Legault, S. J. F. Vincent, J. Greenblatt, R. Konrat and L. E. Kay, *Journal of the American Chemical Society* **1997**, *119*, 6711-6721.
